# Development of an Automatic Computer Program to Determine the Optimal Dental Implant Size and Position for Fibula Free Flap Surgery

**DOI:** 10.3390/cmtr18040046

**Published:** 2025-10-25

**Authors:** Ming Yan Cheung, Ankit Nayak, Xing-Na Yu, Kar Yan Li, Yu-Xiong Su, Jingya Jane Pu

**Affiliations:** 1 Division of Oral and Maxillofacial Surgery, Faculty of Dentistry, The University of Hong Kong, 34 Hospital Road, Sai Ying Pun, Hong Kong, China; ccmy8@connect.hku.hk (M.Y.C.); ankitnayak42@gmail.com (A.N.); rinayu812@gmail.com (X.-N.Y.); 2 School of Advance Engineering, UPES, Dehradun 248007, India; 3 Clinical Research Centre, Faculty of Dentistry, The University of Hong Kong, Hong Kong, China; skyli@hku.hk

**Keywords:** computer-assisted surgery, Jaw reconstruction, fibula free flap, simultaneous dental implant, oral oncology, head and neck cancer, dental rehabilitation

## Abstract

Computer-assisted surgery (CAS) and virtual surgical planning (VSP) have transformed jaw reconstruction, allowing immediate insertion of dental implants during surgery for better rehabilitation of occlusal function. However, traditional planning for optimal location and angulation of dental implants and fibula relies on experience and can be time-consuming. This study aimed to propose a function-driven workflow and develop an automatic computer program for optimal positioning of simultaneous dental implants and fibula segments. A customized computer program was developed using MATLAB. Computed tomography (CT) of the lower limbs of ninety-one Southern Chinese individuals was retrieved and cross-sections of three-dimensional (3D) fibula models were comprehensively investigated for implant installation. Our research proves that the accuracy of the program in identifying the anatomical orientation of the fibula was 92%. The ideal location, angulation and length of implant could be automatically generated based on any selected implant diameter, with a surgical feasibility of 94%. To the best of our knowledge, this is the first study to develop and validate a customized automatic computer program for osseointegrated implant design in fibula flap surgery. This program can be incorporated into the current workflow of CAS to further the development of reliable and efficient surgical planning for function-driven jaw reconstruction.

## 1. Introduction

**Figure 1:**
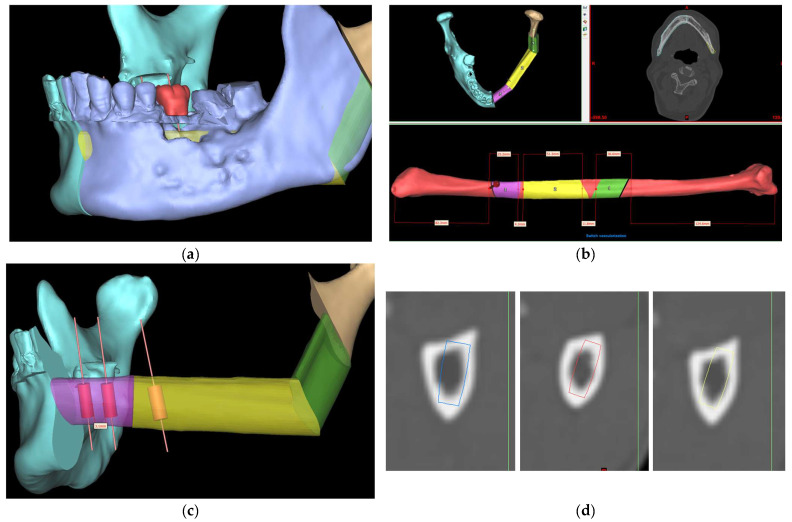
Computer-aided virtual surgical planning workflow: (**a**) CT data of the recipient jaw and donor fibula were segmented to construct the 3D models; (**b**) virtual reconstructive surgeries were conducted using ProPlan CMF 3.0 and the positions of fibula segments were optimized to provide an excellent contouring of defects; (**c**) implants were planned digitally on ideal positions based on the pre-existing dentition; and (**d**) implant positions were checked in the CT lower limbs to ensure bi-cortical engagement and no threads exposure.

Defects of the maxilla and mandible as a result of tumor resection, trauma, bony necrosis after radiation, or anti-resorptive drugs often lead to significant aesthetic and functional deficits [1]. Hidalgo first described the use of a fibula free flap (FFF) in the maxillofacial region for mandibular reconstruction in 1989 [2]. FFF reconstruction with simultaneous dental implant placement has become a pivotal approach for the functional reconstruction of jaw defects following resection of benign or malignant tumors, allowing the functional rehabilitation of chewing, swallowing, speech, and aesthetics [3]. Conventional freehand mandibular reconstruction could be challenging and time-consuming as it greatly relies on the surgeon’s experiences with ‘trial and error’ during the operation [4,5,6]. Positioning of fibula segments using the conventional approach could make dental implant placement technically challenging or impossible. Computer-assisted surgery (CAS) and Virtual Surgical Planning (VSP) have transformed jaw reconstruction, enabling the immediate insertion of dental implants during surgery for improved rehabilitation of occlusal function [7,8,9,10,11]. Our center has previously proposed and adopted the novel “surgeon-dominated” approach to the design of 3D-printed patient-specific surgical plates [12]. Figure 1 demonstrates the current digital workflow at our center. The size of the selected implant depends on the height and width of available bone [13]. Although the fibula looks like a uniformly shaped long bone, determining the ideal implant position and angulation at the fibula segments during VSP could be time-consuming as its cross-sectional shape and dimension vary along its length [11,14]. ‘Trial and error’ and frequent switching between the planning software and CT scan were often needed to ensure that there would be enough bone surrounding the implant to optimize its stability and survival.

Fibula free flap surgery with simultaneous dental implant placement is now routinely performed for functional jaw reconstruction in our center. However, additional time was needed for preoperative planning, especially in determining the orientation of dental implants and fibula segments to restore aesthetics and function after ablative surgery. To address this issue, a precise evaluation of bone dimensions and morphology is essential [15]. Previous cadaveric anatomical studies of the fibula have shown that the length of the harvested fibula and the bone available for implant placement vary between ethnicities [4,14,16,17,18]. And more recently, a CT scan-based analysis of fibula anatomy was carried out to find out the optimal site for implant installation in the fibula; however, the sample size of 20 patients was small [19]. Another limitation of previous studies was that only a linear measurement was recorded from the anterior surface to the posterior aspect of the fibula cross-section, with little attention paid to the anatomical orientation, surgical feasibility, or width of the dental implant within the cross-section.

Although CAS showed increased intraoperative efficiency with a reduced total operative time, pre-operative computer planning could be time-consuming [4]. In the case of fibula implant planning, the process of choosing the appropriate implant dimension could be tedious as it often requires ‘trial and error’ to ensure good angulation of dental implants, bi-cortical engagement, and enough bony collar over the entire implant without any thread exposure, thereby improving its stability and survival. Existing commercial planning software (for example, Materialise ProPlan CMF 3.0 and Materialise Mimics Enlight CMF 7.0) is mainly designed for virtual surgical planning for jaw reconstruction rather than dental implant planning. Therefore, a fully automatic program is required to determine the optimal size and location of dental implants and fibula segments while respecting the anatomical considerations of a fibula free flap harvest. This will pave the way towards automatic placement of dental implants during VSP to provide a reliable and efficient solution for functional jaw reconstruction with a fibula free flap.

The aim of the current study is to describe the development and verification of an automatic program to determine the optimal size and angulation of simultaneous dental implants in functional jaw reconstruction of jaw defects with a fibula free flap.

## 2. Materials and Methods

### 2.1. Study Population

This study was approved by the Institutional Review Board of the University of Hong Kong/Hospital Authority of Hong Kong West Cluster (IRB: UW 24-121). Patients planned for fibula free flap jaw reconstruction in the Department of Oral and Maxillofacial Surgery, Queen Mary Hospital, Hong Kong, from January 2018 to February 2023 were retrospectively recruited. Ninety-one CT scans of the lower limbs were available and retrieved for analysis. CT angiography (CTA) of the lower extremity was taken on a General Electric Revolution System (GE Healthcare, Chicago, IL, USA) with a 1.25 mm thickness with bolus tracking at the popliteal artery at the arterial phase. The patients’ demographic and clinical information were obtained from medical records.

Inclusion criteria included the following: (a) Chinese patients aged 18 years or above at the time of diagnosis; (b) patients diagnosed with oral and maxillofacial pathology from January 2018 to February 2023 and indicated for fibula free flap reconstruction with or without simultaneous implant rehabilitation; and (c) a CT scan of the lower limbs was performed and available for analysis.

Exclusion criteria included the following: (a) patients younger than 18 years old at the time of diagnosis; (b) ethnicity that was not Chinese; (c) fibula free flap was not required for the reconstruction of the defect after tumor resection; and (d) patients who were unable to undergo a preoperative CT scan of the lower limbs.

### 2.2. Image Processing for Measurements

The CTA of lower limbs were exported as Digital Imaging and Communications in Medicine (DICOM) file format, which were then imported into ProPlan CMF 3.0 software (Materialize, Leuven, Belgium). Images were segmented by radiodensity to rebuild the 3D virtual models of the fibula bone (CT threshold values: Min. 226; Max. 3071). The models were exported as stereolithography (STL) data and analyzed using a customized computer program created by our team, as described in detail below.

### 2.3. Customized Computer Program

#### 2.3.1. Identifying the Anterior Aspect of the Fibula

MATLAB platform R2024a was used to develop and run the customized computer program. The computer program began by calculating the central axis of the bone. The bone was repositioned so that its lowest point was at the origin (0, 0, 0). The bone point cloud was aligned with the *z*-axis, maintaining the vertical orientation during slicing. The algorithm identified the anterior direction of the fibula using the most protruded part, which involved calculating the distance of each point from the central axis, identifying the furthest group of points, and computing their average vector to represent the anterior direction of the bone.

**Figure 2:**
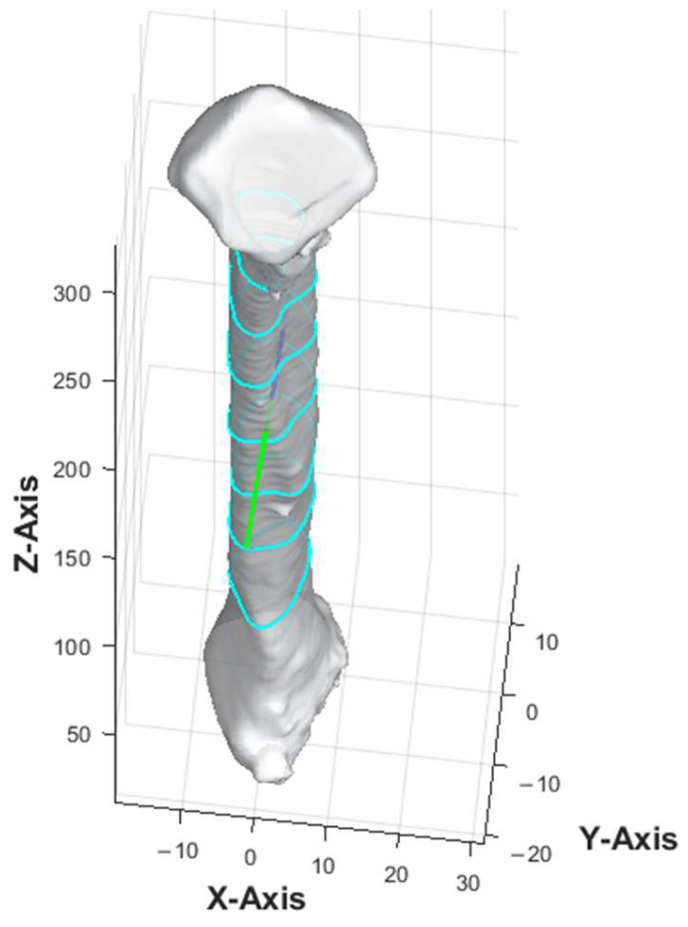
Three-dimensional model of fibula. The contour, as illustrated by the light blue lines, represented the cross-section of the fibula model at different slice heights. Green arrow represented the anterior aspect of fibula model.

The anterior aspect of the 3D fibula model was indicated by a green arrow in Figure 2. Once the anterior vector was calculated, the bone point cloud was rotated to align the anterior vector along the *y*-axis (0, 1, 0), ensuring a consistent orientation of the anterior direction relative to the coordinate system. The threshold for defining the protrusion and the averaging method were determined after iterative testing on multiple fibula models to ensure consistent identification of the anterior vector.

#### 2.3.2. Cross-Section of Fibula at Different Heights

**Figure 3:**
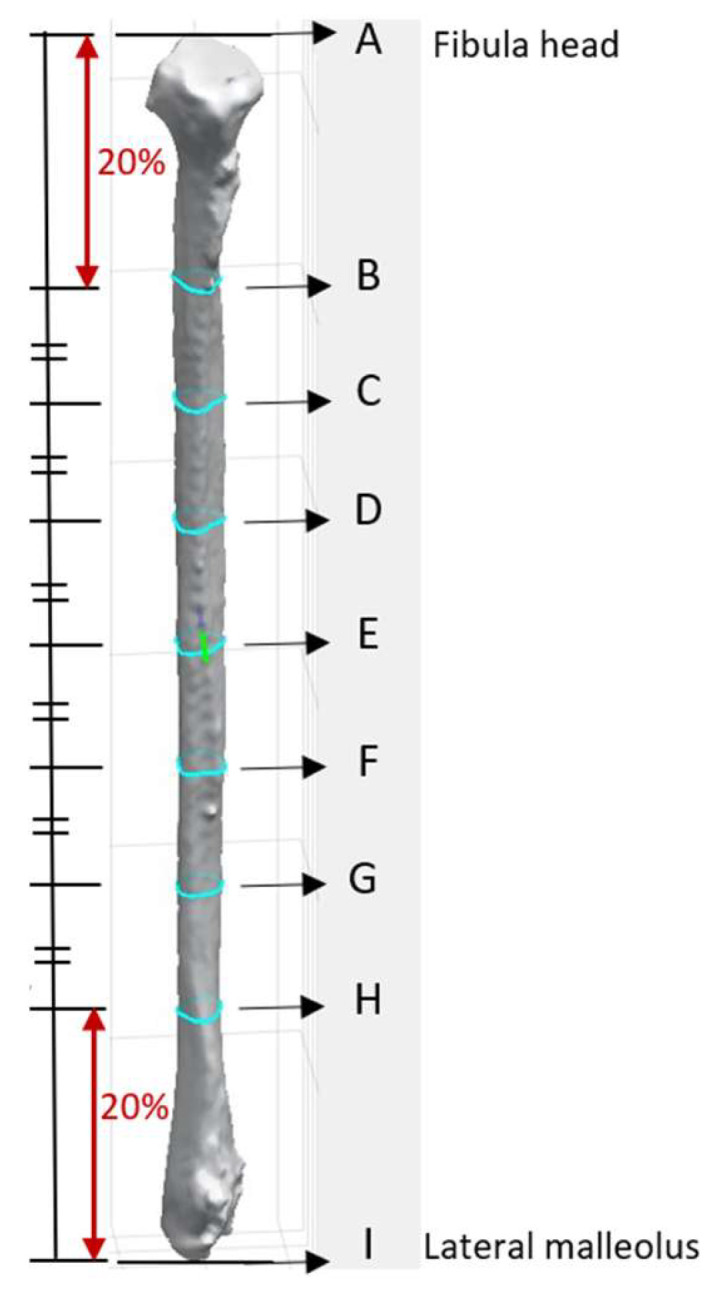
Position of the cross-section of the fibula bone model.

The apex of the fibula head was denoted as point A, and that of the lateral malleolus was point I (Figure 3). The bone was then sliced at seven different heights. The 8 cm distances measured from the apex of the fibula head and that of the lateral malleolus were not generally used for harvesting to maintain the integrity of the knee and ankle [13,16]. These areas each correspond to approximately 20% at the top and bottom of the fibula length and were excluded from the analysis. The remaining 60% of the fibula length was divided into six equal segments to obtain the cross-section geometry along the fibula. Each of the dividing points from the apex of the fibula head was denoted B, C, D, E, F, G, and H as illustrated in Figure 3. This provided a detailed representation of the bone’s structure at various heights, enabling a comprehensive understanding of its geometry and features. The number of segments and the distance between each layer were user-defined parameters.

**Figure 4:**
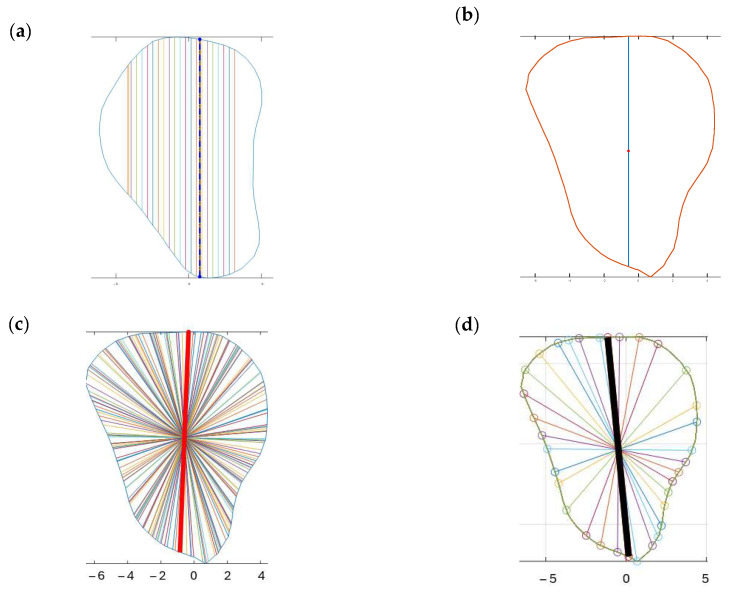
Algorithm for references lines: (**a**) algorithm for red line—grid line with the maximum length; (**b**) algorithm for green line—demonstrating a vertical line that passes through the geometric center of the contour; (**c**) algorithm for blue line—the longest line was highlighted among the different lines that pass through the geometric center of the contour; (**d**) algorithm for black line—blue line was rotated around its midpoint at constant intervals, the highlighted line in black was the identified line for drawing the rectangle.

Four different-colored reference lines represented the outputs of four distinct algorithms, which helped differentiate their outputs and facilitate comparison and analysis of the results. Each algorithm and its contribution to the overall analysis of the bone geometry are detailed in the following sections:

Red line: A set of 100 equidistant gridlines was drawn parallel to the *y*-axis (anterior direction), as illustrated in Figure 4a. Among these gridlines, the one with the maximum length was identified as the central axis of the red rectangle.

Green line: The algorithm calculated the geometric center point of the contour and drew a vertical line through this center point, parallel to the *y*-axis, as illustrated in Figure 4b. Since the anterior direction is aligned to the *y*-axis (as described in Section 2.3.1), the vertical line that is parallel to the *y*-axis represents the thickness of the bone in the anterior direction.

Blue line: It computed the average position of all vertices in the contour and drew lines from each point situated on the contour’s boundary that pass through the contour’s average position (geometric center) and end on the opposite side of the average point, as illustrated in Figure 4c.

Black line: In this algorithm, the blue line served as a reference line and was continuously rotated around its midpoint, which referred to the middle of the line itself rather than the center of the entire contour. The rotation was performed in 1° increments, and each orientation was analyzed to determine the configuration that generated the largest possible rectangle of a predetermined fixed width within the polygon. The 1° step size was selected following pilot testing with increments ranging from 0.5° to 5°, where it offered the best balance between accuracy and computational efficiency. This parameter remains user-adjustable, allowing flexibility in defining the rotation resolution. The line with the optimal orientation was then used to construct the final rectangle, as illustrated in Figure 4d.

**Figure 5:**
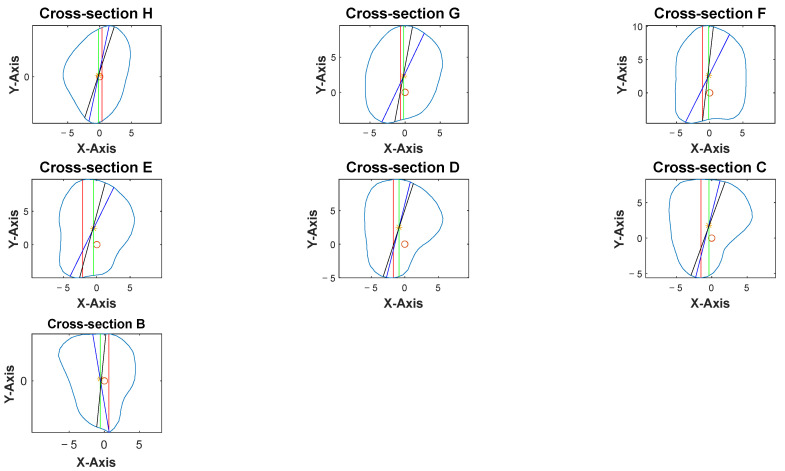
Software interface demonstrating the seven fibula cross-sections with four reference lines. Blue contour represented the outline of each cross-section from point B to H. The *X*-axis represented the anterior aspect of the fibula.

Cross-sections at the potential harvesting sites (points B to H) were then analyzed, alongside the generation of red, green, blue, and black reference lines (Figure 5).

#### 2.3.3. Rectangle Plot

Based on the coordinates of the four reference lines and a user-defined value for the width of the rectangle, four colored rectangle plots would be automatically generated using the corresponding algorithms. Each rectangle represented the greatest dimension of dental implants with the proposed angulation.

### 2.4. Clinical Considerations

#### 2.4.1. Implant Systems

**Figure 6:**
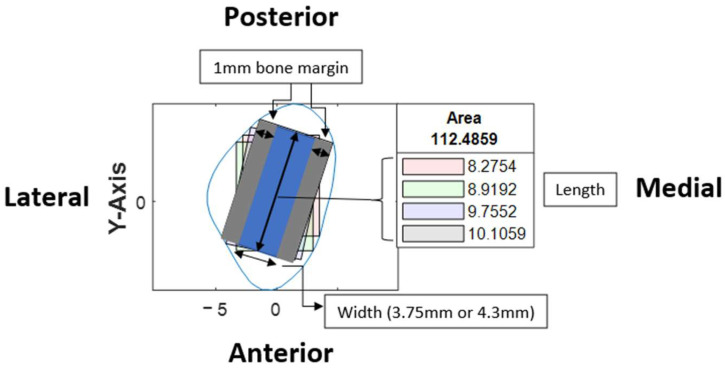
Illustration of rectangles within a cross-section and the orientation of the cross-section.

**Figure 7:**
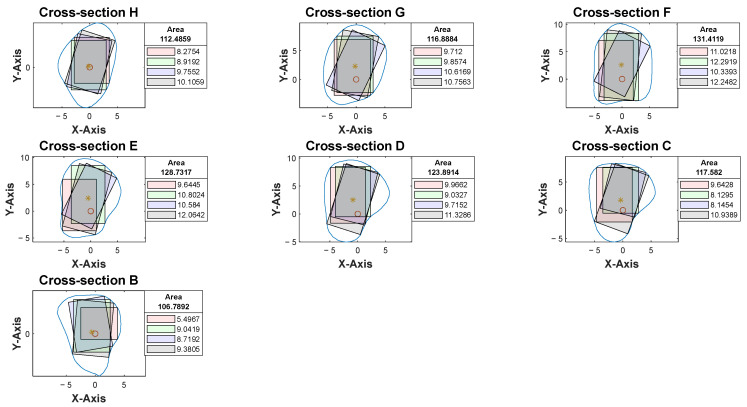
Software interface demonstrating cross-section of fibula segments and four colored rectangles, each representing the different angulation and implant length generated by the four distinct algorithms. The length of each rectangle was automatically generated and shown in the textbox.

At our center, the standard dental implant system used for functional jaw reconstruction is the Nobel Parallel Conical Connection TiUltra (Nobel BioCare AB, Göteborg, Sweden). The implant dimensions commonly used were narrow (Ø3.75 mm) and regular (Ø4.3 mm) platforms, with a length of 8.5 mm, 10 mm, 11.5 mm, and 13 mm. However, this number could be easily adjusted in the program with a different implant system. The width of the rectangle represented the user-defined implant diameter with an additional 1.0 mm bony collar included on both sides of the implant (lateral and medial aspects) to ensure no implant thread exposure through the bone [18,19,20]. Hence, the widths of rectangles for regular and narrow implants were 6.3 mm and 5.75 mm, respectively (Figure 6). This was used as the input value to allow the program to automatically generate the four colored rectangles and calculate the optimal angulation and maximum implant length at sections B, C, D, E, F, G, and H (Figure 7).

#### 2.4.2. Anatomical Considerations for the Fibula Free Flap

The length of the rectangle corresponded to the maximal length of implant possible at each cross-section, with consideration of the surgical feasibility (i.e., from the anterior or lateral aspect of the fibula to the posterior border) and avoidance of the postero-medial portion to prevent interruption to the blood supply from the pedicle.

### 2.5. Workflow

With the use of MATLAB software R2024a, STL files of the fibula model were imported and analyzed automatically by the program. Each STL file would generate three popup windows, including the 3D bone model (Figure 2), cross-sections with four colored reference lines (Figure 5), as well as the cross-sections with four colored rectangles at seven different heights representing the ideal angulation and length of dental implant for each algorithm (Figure 7). A spreadsheet file would be generated automatically to allow direct observation and comparison of the maximum length of rectangles.

The orientation of fibula bone models was verified by two assessors (M.Y.C. and X.-N.Y.) to evaluate the accuracy level of the program in identifying the anterior edge of the fibula, as this determines the correct insertion angle of the dental implants. The two assessors would then go through the cross-section analysis for each bone model to verify the anatomical considerations of implant insertion in a fibula free flap and select the rectangle with the greatest length. The inter-rater reliability was assessed for each measurement. A third assessor was involved when the discrepancies were out of range.

### 2.6. Statistical Analysis

Statistical analysis was performed using IBM SPSS Statistics Version 28. The two-way random intraclass correlation coefficient was used to determine the level of agreement between two raters when determining the orientation of the fibula and analyzing the best angulation and dimension of the implant at each cross-section.

## 3. Results

### 3.1. Patient’s Demographic Background

The demographic data and disease characteristics of patients are presented in Table 1. Of the 91 Chinese patients recruited, 52 (57%) were male and 39 (43%) were female. Patients’ ages ranged from 20 to 86 years, with a mean of 58.4 ± 16.7 years. No significant age difference was found between genders (*p* = 0.882). The majority of the lesions were found in the mandible (77%) and were malignant in nature (68%).

### 3.2. Program Accuracy

**Figure 8:**
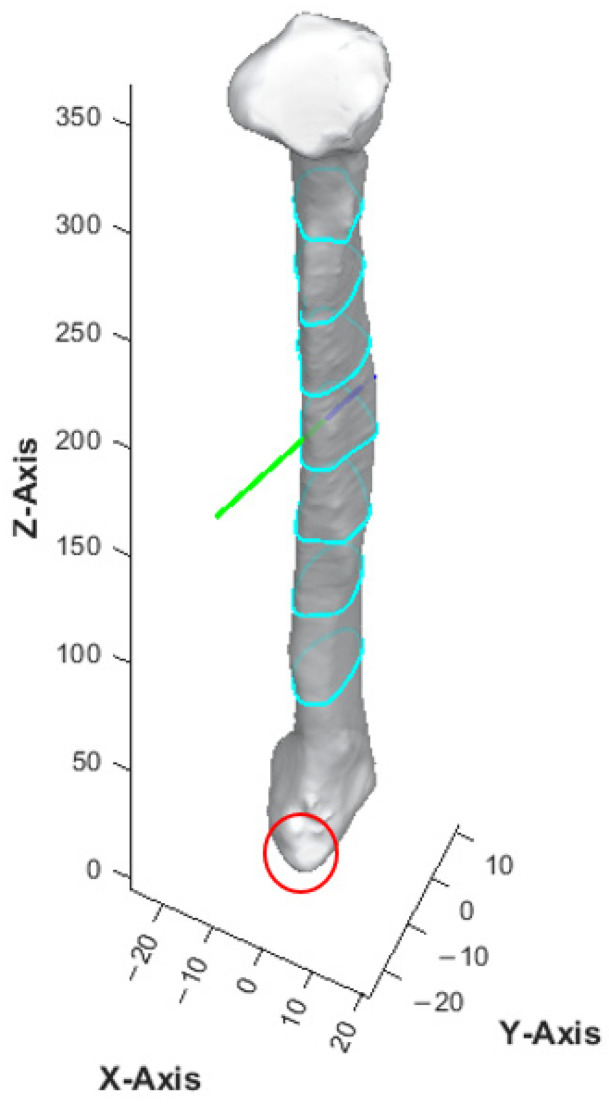
Surface model of fibula bone. The anterior surface of lateral malleolus is circled in red. The green and dark blue lines represented the anterior and posterior aspects of the fibula model respectively.

The accuracy level in identifying the orientation and anterior edge of the fibula model was verified before proceeding with cross-sectional analysis. After multiple rounds of optimization of the computer program with human verification, no to minimal manual adjustments were required in orienting the fibula models in the current software version. With the most protruded part method as described earlier, the anterior surface of the lateral malleolus, as highlighted in red in Figure 8, was found to be a reliable benchmark for identifying the inferior and anterior direction of the fibula model. Among the four colored rectangles, the black rectangle demonstrated the maximum length with surgical feasibility in 94% of the cross-sections (Table 2).

### 3.3. Inter-Rater Agreement Measures

**Figure 9:**
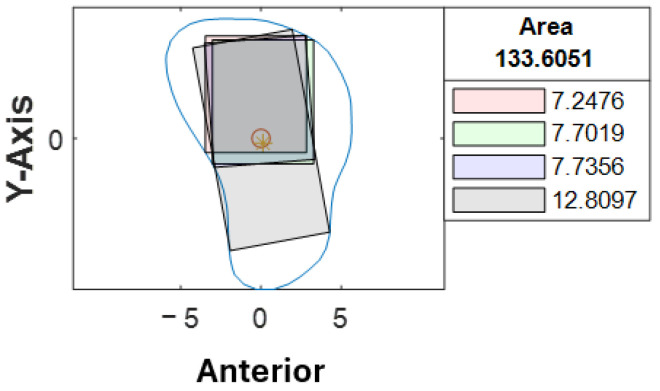
Example of irregular fibula cross-section.

**Figure 10:**
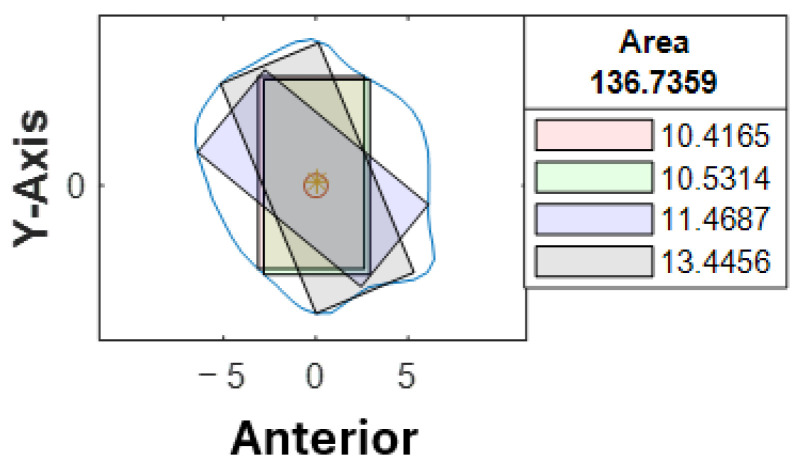
Different implant angulations.

**Figure 11:**
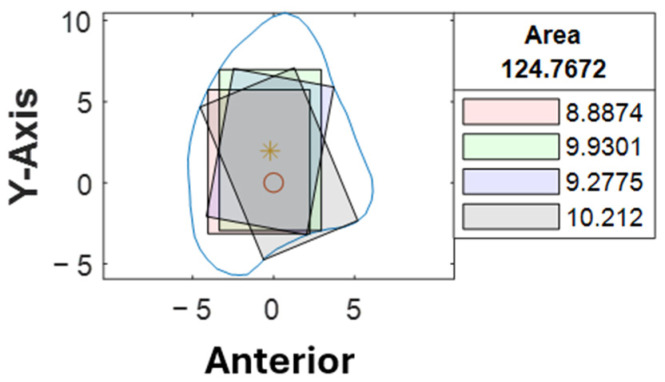
Black rectangle out of contour.

A second assessor (X.-N.Y.) conducted the same analysis independently using the customized computer program. Inter-rater agreement was assessed on all STL bone models. The intraclass correlation coefficient was 0.963 with a 95% confidence interval, indicating that the level of agreement and consistency between the two raters when analyzing the best angulation and dimension of the implant at each cross-section was excellent. Among 1274 cross-sections, a different colored rectangle was chosen between the raters in 107 cross-sections (8%) when considering both the maximum possible length of implants and surgical feasibility. Table 3 lists the number of measurements that were not in agreement concerning the location of the cross-section. Cross-section B-C, D-F, G-H correspond to the proximal, middle and distal segments respectively. More discrepancy (52%) was noted towards the proximal end of the fibula, where a more irregular contour was likely to be observed (Figure 9). 48% of the discrepancies had a difference of less than 1.0 mm (as listed in Supplementary Material, Table S1). 79% (85/107) of the disagreements were due to different preferences when determining the ideal implant angulation. Both measurements were surgically feasible; however, one may prefer to insert the implant from the anterior aspect of the fibula (as shown by the black rectangle in Figure 10) and the other from a relatively more lateral aspect (as indicated by the blue rectangle in Figure 10). For the remaining 21% (22/107) of the cases, one may consider that it remains surgically feasible when a small portion of the rectangle (<1.0 mm) exceeded the contour of the cross-section (Figure 11).

Among the disagreements, 7% of the differences (7/107) were out-of-range based on 95% limits of agreement (as highlighted in Table S1). The location of the fibula segment with the out-of-range differences was recorded, and almost all of those were located towards the proximal end of the fibula (Table S1). A third assessor (J.J.P.) was invited for the assessment. For 5 of them, the black rectangles presented with a slightly insufficient bony collar due to the concave shape of the fibula bone (Figure 9). The surgical feasibility was confirmed by the third assessor. For the other two cases, the cross-section of the fibula presented with a significantly bigger dimension in the mediolateral dimension than in the anteroposterior dimension. The shorter implants were selected for anatomical safety.

## 4. Discussion

Fibula free flap with simultaneous dental implant placement is now routinely performed for functional jaw reconstruction in our center. However, additional time was needed for preoperative planning, especially in determining the orientation of dental implants and fibula segments, to restore aesthetics and function after ablative surgery. To the best of our knowledge, this is the first study to determine the optimal size and angulation of dental implants using an automatic computer program, while incorporating surgical considerations when harvesting a fibula free flap for jaw reconstruction.

To ensure surgical feasibility, the anterior aspect of the fibula was automatically identified using the program developed, hence the direction of implant installation. Our research has proved that the accuracy and reproducibility of the program in identifying the anterior surface of the fibula were as high as 92%. The ideal location, angulation, and maximum length of implant could be automatically generated based on the selected implant diameter. In addition, the implants’ ideal position according to prosthetic considerations predetermines the position of the fibula segments for bony reconstruction [19]. This is an important step towards automatic function-driven fibula reconstruction of the jaw.

In the current study, the specific algorithm for generating the black rectangle proved to be the most effective in representing the ideal angulation with maximum implant length and surgical feasibility in 94% of the cases studied. Based on this significant finding, further efforts could be made to streamline and improve this specific algorithm, aiming to convert implant selection and planning into a fully automated procedure that requires minimal manual adjustments. At the same time, the computer coding could be easily edited to remove the other three algorithms to allow a more focused interpretation using the black algorithm. The automated program could potentially reduce virtual implant planning time, especially in complex cases [21]. To the best of our knowledge, there is no similar automated function in currently available commercial virtual surgical planning software yet.

The inter-rater agreement of 0.963 indicated that rater-related variance and random error were minimal, meaning the raters’ measurements were nearly identical across most cases. It demonstrated that the computer program was user-friendly and could be relied upon to pre-determine the ideal implant angulation based on cross-sectional analysis. Although disagreements (8%) were present between the raters, and 7% of them were out of range, these contributed to 0.5% of all the cross-sections analyzed. The third rater was involved when the discrepancies between the first and second raters were out of range; the results were in line with the first rater. According to our observations, the proximal end of the fibula tends to have a more irregular or triangular cross-sectional shape. Therefore, most of the discrepancies were noted towards the proximal segments, where a slight difference in implant angulation could result in a greater difference in maximum implant length (Figure 9).

We concluded that there were two main reasons for the discrepancies. Different surgeons might have different preferences for ideal implant angulation (Figure 10). One might prefer to insert from the anterior or lateral aspects of the fibula to maximize the possible implant length, while both are surgically acceptable. Secondly, there might be a different opinion on the concept of surgical feasibility [13]. A small portion of the rectangle (<1.0 mm) exceeding the contour of the cross-section might be considered to be surgically acceptable, as the final implant length was determined by subtracting a 1.0 mm bony collar at the apical portion from the maximum length of the rectangle [18]. Furthermore, when referring to the actual clinical application, a smaller implant diameter could be considered in the above scenario to avoid possible implant thread exposure. Implant placement might be avoided in areas with irregular cross-sectional shapes. The cross-sectional analysis allowed clinicians to have a better understanding of the fibula morphology, hence identifying the best segment for implant placement. When the program produces a result as illustrated in Figure 9, this may help to alert the surgeon, and further evaluation should be carried out, as those segments might not be suitable for implant placement. This is why an automated computer program specifically designed for implant planning would be beneficial in VSP for fibula reconstruction.

As with all computer programs or software, regular updates are required to further improve the computer coding and its functionality. This is particularly important when the program is applied to a larger study population, as it would likely require updates to accommodate the anatomical variations in the population for a more reliable and accurate analysis. With significant advances in artificial intelligence and machine learning, this technology can be further integrated into the workflow of automatic virtual surgical planning for functional jaw reconstruction.

Regarding the study limitations, apart from its retrospective nature, switching between different software might be necessary during VSP. Current commercial software is not open source and therefore not available for the integration of third-party developer programming. We recommend using this customized computer program to first analyze the fibula morphology and identify the ideal fibula segments for implant placement before proceeding with the routine VSP with commercial software. To improve clinical integration of the program, the aim is to develop CAS planning software that incorporates the algorithm for implant planning. This is a major step towards a fully integrated automatic surgical planning platform. On the other hand, although the computer program worked for the majority of the cases (92%), for fibula with irregular morphology, especially towards the proximal end, greater differences in maximum possible implant length generated by the algorithms were noted, hence resulting in out-of-range differences in the very limited number of extreme cases. In actual clinical situations, these areas might not be ideal for implant planning. In most of our cases, the proximal end of the fibula was often discarded for the pedicle length. This automatic program currently acts as an aid in analyzing the fibula cross-section with the consideration of the ideal dimension and angulation of dental implants. Surgeons should make the final clinical decision to ensure intraoperative safety, especially in those extreme cases.

The current study mainly focused on the development and streamlining of the program; clinical validation would be an outcome for future studies. Further prospective clinical studies incorporating this customized computer program are needed to validate the protocol. On the other hand, clinical feasibility or accuracy could be validated by using existing VSP software pre-operatively to check whether the planned implant location with the program could be safely performed during the surgery.

## 5. Conclusions

This current study describes the development and verification of an automated program to determine the optimal size and angulation of dental implants in functional jaw reconstruction using a fibula free flap. This customized automatic computer program is readily available. It can be incorporated into the current guided implant design workflow to advance developments in function-driven fibula reconstruction of the jaw. Further prospective clinical studies are warranted to validate the clinical performance of the program. This will pave the way towards automatic placement of dental implants during VSP to provide a reliable and efficient solution for functional jaw reconstruction in fibula free flap surgery.

## 6. Patents

Title of invention: Method and system for automatic identification of a dental implant position on a bone. US 63/754,065. Filing date: 5 Feb 2025.

## Figures and Tables

**Table 1:** Patient demographics and reconstruction characteristics.

Characteristic	Male (n = 52)	Female (n = 39)	*p* Value
Age (mean)	58.6 ± 17.7	58.2 ± 15.4	0.882 ^b^
**Lesion type**			
Benign	21.2% (11/52)	25.6% (10/39)	0.857 ^c^
Malignant	69.2% (36/52)	66.7% (26/39)	
Others **^a^**	9.6% (5/52)	7.7% (3/39)	
**Defect site**			
Maxilla	19.2% (10/52)	28.2% (11/39)	0.315 ^c^
Mandible	80.8% (42/52)	71.8% (28/39)	

^a^ Others included secondary reconstruction and osteoradionecrosis; ^b^ This *p*-value was calculated by means of the Mann–Whitney U test; ^c^ This *p*-value was calculated by means of the chi-square test.

**Table 2:** Algorithm selected in fibula cross-sections.

Rectangles Generated by Algorithm	Number of Cross-Sections
Black	94% (1195/1274)
Green	3% (41/1274)
Red	2% (22/1274)
Blue	1% (16/1274)

**Table 3:** Number of measurements not in agreement between raters.

		Cross-Section						
		B	C	D	E	F	G	H
Female	5.75 mm	4	2	**4**	**1**	**4**	3	1
	6.3 mm	6	7	**8**	**3**	**3**	5	1
Male	5.75 mm	13	6	**3**	**3**	**2**	0	1
	6.3 mm	15	2	**5**	**3**	**0**	1	1
Total		38	17	**20**	**10**	**9**	9	4
Percentage		36	16	**19**	**9**	**8**	8	4

## Data Availability

The original data presented in the study are openly available in FigShare at DOI 10.6084/m9.figshare.29413136.

## Supplementary Materials

The following supporting information can be downloaded at https://www.mdpi.com/article/10.3390/cmtr18040046/s1. Table S1: Table showing the differences in measurements between raters.

## Abbreviations

The following abbreviations are used in this manuscript:

CAS	Computer-assisted surgery
CT	Computer tomography
CTA	Computer tomography angiography
DICOM	Digital Imaging and Communications in Medicine
FFF	Fibula free flap
STL	Stereolithography
VSP	Virtual surgical planning
3D	Three-dimensional
Ø	Diameter
