# Supplementary material for: Development of an Automatic Computer Program to Determine the Optimal Dental Implant Size and Position for Fibula Free Flap Surgery
Source: Craniomaxillofac Trauma Reconstr. 2025 Oct 25;18(4):46. doi: 10.3390/cmtr18040046 (PMC12641722; doi:10.3390/cmtr18040046)
Supplement: Supplementary file 1 [file cmtr-18-00046-s001.zip › cmtr-3737040-supplementary.pdf]

Table S1. Table showing the differences in measurements between raters.

Number	Measurements (mm) Rater 1 (MY.C)	Rater 2 (XN.Y)	Absolute Difference	Difference	Location	Rater 3 (JJ.P)
1	10.49	8.98	1.51	1.51		
2	13.06	13.38	0.32	-0.32		
3	10.20	10.64	0.44	-0.44		
4	8.08	9.50	1.42	-1.42		
5	9.33	5.80	3.53	3.53		
6	11.66	12.49	0.83	-0.83		
7	12.55	12.64	0.09	-0.09		
8	11.02	11.05	0.03	-0.03		
9	9.06	6.05	3.01	3.01		
10	6.08	5.78	0.30	0.30		
11	9.60	8.47	1.13	1.13		
12	7.60	7.45	0.15	0.15		
13	15.19	15.22	0.03	-0.03		
14	6.65	5.75	0.90	0.90		
15	7.63	9.87	2.24	-2.24		
16	5.75	5.83	0.08	-0.08		
17	7.75	9.45	1.70	-1.70		
18	8.63	9.46	0.83	-0.83		
19	11.74	11.88	0.14	-0.14		
20	10.12	8.79	1.33	1.33		
21	7.53	6.84	0.69	0.69		
22	11.29	11.34	0.06	-0.06		
23	9.14	7.63	1.51	1.51		
24	10.53	10.60	0.07	-0.07		
25	11.05	10.39	0.66	0.66		
26	13.74	11.56	2.18	2.18		
27	10.40	9.52	0.88	0.88		
28	10.38	10.62	0.24	-0.24		
29	9.06	5.33	3.73	3.73		
30	8.29	8.33	0.04	-0.04		
31	9.57	10.28	0.71	-0.71		
32	6.08	5.24	0.84	0.84		
33	7.03	10.25	3.22	-3.22		
34	8.01	8.02	0.01	-0.01		
35	8.53	8.60	0.07	-0.07		
36	9.94	6.56	3.37	3.37		
37	11.50	11.56	0.06	-0.06		
38	14.30	14.42	0.12	-0.12		
**39**	**12.81**	**7.74**	**5.07**	**5.07**	**D**	**12.81**
40	5.65	5.11	0.54	0.54		
**41**	**13.33**	**6.80**	**6.53**	**6.53**	**C**	**13.33**
42	9.77	7.84	1.93	1.93		
43	6.30	7.24	0.94	-0.94		

44	7.85	8.70	0.85	-0.85		
45	11.06	10.42	0.64	0.64		
46	11.41	9.98	1.43	1.43		
47	5.19	4.30	0.89	0.89		
48	14.26	11.04	3.22	3.22		
**49**	**9.31**	**4.60**	**4.71**	**4.71**	**B**	**9.31**
50	6.06	4.33	1.73	1.73		
51	8.01	9.10	1.09	-1.09		
52	11.45	11.61	0.16	-0.16		
53	13.13	12.19	0.94	0.94		
54	13.87	12.65	1.22	1.22		
55	12.55	12.24	0.31	0.31		
56	13.95	13.88	0.07	0.07		
57	8.11	10.33	2.22	-2.22		
58	12.25	13.54	1.29	-1.29		
59	6.97	8.83	1.86	-1.86		
60	19.19	19.08	0.11	0.11		
61	12.71	13.08	0.37	-0.37		
62	14.67	13.14	1.53	1.53		
**63**	**12.74**	**6.24**	**6.50**	**6.50**	**C**	**12.74**
64	11.48	11.59	0.11	-0.11		
65	17.07	15.88	1.19	1.19		
66	14.59	12.16	2.43	2.43		
67	11.72	12.51	0.79	-0.79		
**68**	**12.10**	**17.84**	**5.74**	**-5.74**	**B**	**12.10**
69	11.07	12.31	1.24	-1.24		
70	12.41	14.05	1.64	-1.64		
71	12.96	14.72	1.76	-1.76		
72	9.96	11.30	1.34	-1.34		
73	13.45	11.47	1.98	1.98		
74	10.76	11.78	1.02	-1.02		
**75**	**10.30**	**5.75**	**4.55**	**4.55**	**B**	**10.30**
76	11.32	12.40	1.08	-1.08		
77	8.26	8.20	0.06	0.06		
78	11.53	13.03	1.50	-1.50		
79	15.66	14.68	0.98	0.98		
80	9.02	12.66	3.64	-3.64		
81	12.45	11.53	0.92	0.92		
82	10.22	7.96	2.26	2.26		
83	7.19	10.05	2.86	-2.86		
84	9.90	10.36	0.46	-0.46		
85	10.41	8.65	1.76	1.76		
86	6.30	8.45	2.15	-2.15		
87	11.31	12.01	0.70	-0.70		
88	10.46	10.73	0.27	-0.27		
89	14.46	10.41	4.05	4.05		
90	10.92	9.64	1.28	1.28		
91	11.26	11.23	0.03	0.03		

92	16.69	15.41	1.28	1.28		
**93**	**11.84**	**17.34**	**5.50**	**-5.50**	**B**	**11.84**
94	12.59	14.13	1.54	-1.54		
95	10.75	11.94	1.19	-1.19		
96	12.09	13.28	1.19	-1.19		
97	12.42	13.82	1.40	-1.40		
98	9.46	10.87	1.41	-1.41		
99	11.80	10.71	1.09	1.09		
100	10.92	11.30	0.38	-0.38		
101	8.60	7.79	0.81	0.81		
102	7.28	7.08	0.20	0.20		
103	11.18	12.36	1.18	-1.18		
104	11.26	11.35	0.09	-0.09		
105	11.03	11.31	0.28	-0.28		
106	15.06	14.27	0.79	0.79		
107	8.57	12.06	3.49	-3.49		
